# S-Nitrosylation of the virulence regulator AphB promotes *Vibrio cholerae* pathogenesis

**DOI:** 10.1371/journal.ppat.1010581

**Published:** 2022-06-17

**Authors:** Jiandong Chen, Hyuntae Byun, Qianxuan She, Zhi Liu, Karl-Gustav Ruggeberg, Qinqin Pu, I-Ji Jung, Dehao Zhu, Mary R. Brockett, Ansel Hsiao, Jun Zhu

**Affiliations:** 1 Department of Microbiology, Perelman School of Medicine, University of Pennsylvania, Philadelphia, Pennsylvania, United States of America; 2 Department of Microbiology & Plant Pathology, University of California Riverside, Riverside, California, United States of America; University of Maryland, UNITED STATES

## Abstract

*Vibrio cholerae* is the etiologic agent of the severe human diarrheal disease cholera. To colonize mammalian hosts, this pathogen must defend against host-derived toxic compounds, such as nitric oxide (NO) and NO-derived reactive nitrogen species (RNS). RNS can covalently add an NO group to a reactive cysteine thiol on target proteins, a process called protein S-nitrosylation, which may affect bacterial stress responses. To better understand how *V*. *cholerae* regulates nitrosative stress responses, we profiled *V*. *cholerae* protein S-nitrosylation during RNS exposure. We identified an S-nitrosylation of cysteine 235 of AphB, a LysR-family transcription regulator that activates the expression of *tcpP*, which activates downstream virulence genes. Previous studies show that AphB C235 is sensitive to O_2_ and reactive oxygen species (ROS). Under microaerobic conditions, AphB formed dimer and directly repressed transcription of *hmpA*, encoding a flavohemoglobin that is important for NO resistance of *V*. *cholerae*. We found that tight regulation of *hmpA* by AphB under low nitrosative stress was important for *V*. *cholerae* optimal growth. In the presence of NO, S-nitrosylation of AphB abolished AphB activity, therefore relieved *hmpA* expression. Indeed, non-modifiable *aphB*^C235S^ mutants were sensitive to RNS *in vitro* and drastically reduced colonization of the RNS-rich mouse small intestine. Finally, AphB S-nitrosylation also decreased virulence gene expression via debilitation of *tcpP* activation, and this regulation was also important for *V*. *cholerae* RNS resistance *in vitro* and in the gut. These results suggest that the modulation of the activity of virulence gene activator AphB via NO-dependent protein S-nitrosylation is critical for *V*. *cholerae* RNS resistance and colonization.

## Author summary

Beset by constantly evolving armies of potential pathogenic invaders, human hosts deploy a variety of countermeasures, including the production of host-derived toxic compounds nitric oxide (NO) and NO-derived reactive nitrogen species (RNS). In response, pathogens such as *Vibrio cholerae* have developed anti-RNS systems. The flavohemoglobin HmpA is an NO detoxification protein, playing key roles in *V*. *cholerae* RNS resistance. HmpA is transcriptionally activated by the NO sensor NorR. Here we discovered that the *V*. *cholerae* virulence regulator AphB is a repressor of *hmpA*, and a protein S-nitrosylation of AphB serves as an off switch to derepress *hmpA* expression and protects *V*. *cholerae* from nitrosative stress. AphB also promotes *V*. *cholerae* virulence gene expression, and an S-nitrosylation of AphB abolished the production of virulence factors. Using a mouse model, we further explored the importance of the defensive and offensive flexibility afforded by AphB, showing that NO-dependent protein S-nitrosylation is critical for *V*. *cholerae*’s war strategy.

## Introduction

*Vibrio cholerae* is the causative agent of cholera, a severe watery diarrhea disease. There are millions of cases of cholera and thousands of deaths caused by *V*. *cholerae* each year [1]. To colonize and cause disease, *V*. *cholerae* uses sophisticated signal transduction pathways to activate a set of virulence factors [2,3]. The master virulence regulator ToxT controls expression of an array of virulence genes, including genes involved in the synthesis of cholera toxin and toxin-coregulated pilus (TCP). The expression of *toxT* requires ToxR and TcpP, and TcpP is regulated by AphA and AphB. Under anaerobic conditions, *tcpP* expression increases, which is directly regulated by AphB [4,5], a LysR-family protein that is widely conserved in prokaryotes. Under aerobic conditions, one key cysteine residue (Cys235) of AphB is reversibly oxidized. While under low oxygen conditions, AphB Cys235 is reduced, which promotes oligomerization and subsequently enhances AphB activity, thus activating *tcpP*.

In addition to activating virulence genes, *V*. *cholerae* must also coordinate the sequential expression of a series of factors required for defense against host-derived toxic compounds. One of these compounds elevated during infection is nitric oxide (NO), a toxic radical that disrupts the function of proteins containing cysteine residues, enzymes catalyzing iron-dependent reactions, and members of the electron transport chain [6]. Furthermore, NO reacts with other small molecules produced by the immune system to form other toxic reactive nitrogen species (RNS). In the host, NO is generated by acidified nitrite in the stomach and by enzymes of the nitric oxide synthase (NOS) family, such as inducible NOS (iNOS), which is capable of generating large quantities of NO in an inflammatory setting [7]. It has been reported that patients with cholera have an increase in the expression of iNOS in their small intestines [8–10], suggesting that *V*. *cholerae* encounters NO during human infections.

To cope with NO produced during infection, many pathogenic bacteria have evolved mechanisms to convert NO into other, less toxic, nitrogen oxides [6]. In *V*. *cholerae*, HmpA, a member of the flavohemoglobin family of enzymes, plays a key role in resistance of NO by catalyzing the conversion of NO to nitrous oxide or nitrate [11]. In the presence of NO, *hmpA* expression is activated by the NO sensor NorR, a predicted σ^54^-dependent transcriptional regulator [6]. NorR also activates the expression of *nnrS*, which encodes a novel protein that is important for RNS resistance [6]. NnrS does not directly remove NO but instead it protects the cellular iron pool from the formation of dinitrosyl iron complexes (DNICs) [12]. NO also impacts cellular signaling through S-nitrosylation of protein cysteine residues [13]. To investigate how NO affects *V*. *cholerae* protein S-nitrosylation, we performed a proteomic study of *V*. *cholerae* exposed to NO using a modified iodoacetyl isobaric tandem mass tags (iodoTMT) approach [14]. We found that the cysteine 235 residue in the key virulence activator AphB was nitrosylated during RNS exposure. We showed that in the absence of nitrosative stress AphB repressed *hmpA* expression and that nitrosative stress inactivated AphB via protein S-nitrosylation. We also showed that AphB nitrosylation was important for *V*. *cholerae* RNS resistance and pathogenesis *in vitro* and *in vivo*.

## Results

### Proteomic profiling of *V*. *cholerae* protein S-nitrosylation upon RNS exposure

**Fig 1:**
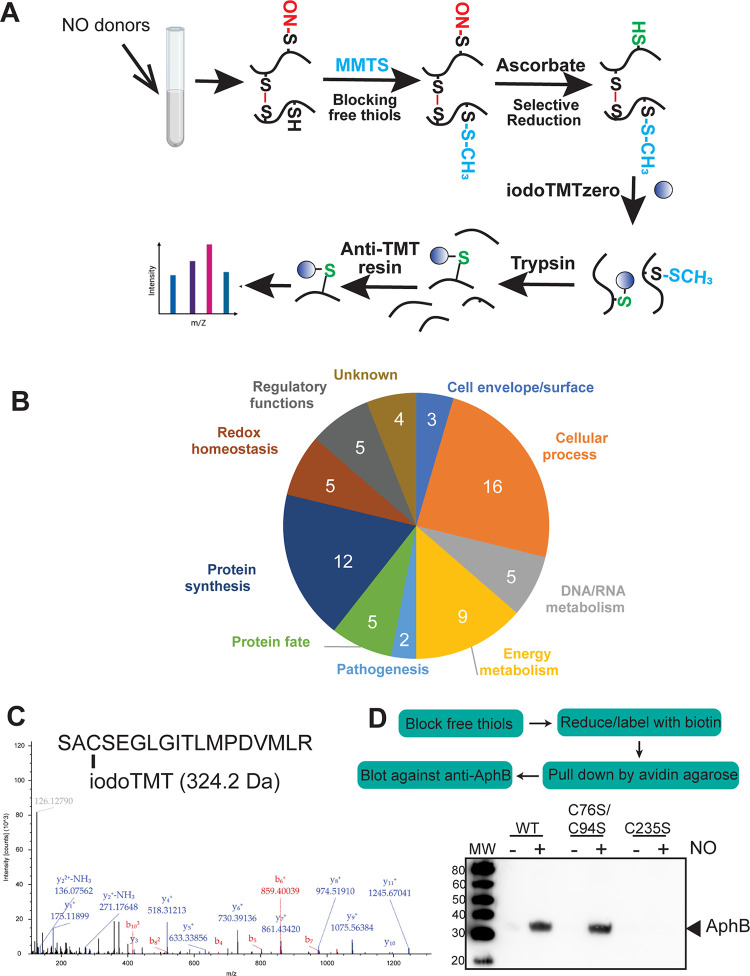
Profiling cysteine S-nitrosylation in the *V*. *cholerae* proteome. **A.** Schematic of the nitrosylated thiol labeling approach. *V*. *cholerae* was grown under the virulence-inducing conditions and subsequently challenged with 100 μM Diethylammonium (Z)-1-(N,N-diethylamino)diazen-1-ium-1,2-diolate (DEA NONOate) for 1 hr. Free thiols in the extracts were blocked. Upon reduction of S-nitrosylated thiols, proteins were labeled with iodoTMTzero. After digestion with trypsin, peptides were captured by an anti-TMT resin. The eluted labeled peptides were then analyzed by LC-MS/MS. **B.** Pie chart showing the distribution of SNO-modified cysteine-containing proteins in *V*. *cholerae* by protein functional categories. **C.** LC-MS/MS spectrum of AphB peptides containing Cys235 TMT modification. **D.** Confirmation of AphB cysteine S-nitrosylation. Cultures of *V*. *cholerae ΔaphB* with P_*BAD*_-*aphB*^WT^ and cysteine→serine mutant derivatives with or without DEA NONOate exposure were subjected to the biotin switch assay [17]. Western blot analysis on avidin agarose-pulled down samples was then performed using the anti-AphB antibody.

To investigate how NO affects *V*. *cholerae* protein S-nitrosylation, we applied a modified iodoTMT approach [14] to identify *V*. *cholerae* protein S-nitrosylation in the presence of NO (Fig 1A). First, we grew *V*. *cholerae* under a virulence-inducing condition (AKI medium) [15] and treated the cells with the NO donor DEA NONOate. The reduced thiols in the extracts were blocked with methyl methanethiosulfonate (MMTS). Nitrosylated thiols were then selectively reduced by sodium ascorbate, and reduced cysteines in proteins were specifically labeled with iodoTMTzero. After digestion by trypsin, peptides were captured by an anti-TMT resin. The eluted labeled peptides were then analyzed by LC-MS/MS. Using this method, we identified 67 proteins containing cysteine S-nitrosylation upon exposure to NO (Fig 1B and S1 Dataset). Several of these identified proteins are important for cellular process, protein synthesis, and energy metabolism implying that S-nitrosylation may have a profound impact on bacterial physiology. A few reactive oxygen species (ROS) resistance proteins, such as thioredoxin peroxidase AhpC, thioredoxin TagD, and glutathione synthetase GssA were also detected, suggesting that there are overlapping roles between ROS and RNS resistance. In mammalian cells, thioredoxins can be nitrosylated and transnitrosylate target proteins [16]. We also detected S-nitrosylation in important virulence factors: toxin co-regulated pilin TcpA and toxin coregulated pilus biosynthesis protein TcpH. Interestingly, we identified S-nitrosylation of the key virulence activator AphB [4,5]. AphB contains three cysteine residues, Cys76, Cys94, and Cys235, and AphB peptides containing Cys235-TMT were detected (Fig 1C). Cys76-TMT was also detected but at a lower abundance than Cys235-TMT (S1 Dataset). In this study, we further investigated the relationship between S-nitrosylation and AphB function.

To confirm S-nitrosylation of AphB, we adapted the biotin-switch assay [17] to detect nitrosylated AphB in cells exposed to NO. The nitrosylated proteins were selectively reduced and labeled with biotin. Biotinylated proteins were subsequently enriched by avidin agarose, and AphB was detected via western blot using anti-AphB antibodies (the flowchart in Fig 1D). We also included cysteine-to-serine mutants of AphB as these mutant proteins could not be modified [5]. Without addition of NO, no AphB was nitrosylated (therefore biotinylated) (Fig 1D). When NO was added, S-nitrosylation occurred in both wildtype AphB and AphB^C76S/C94S^ mutants, but not in AphB^C235S^ mutants (Fig 1D). These data suggest that AphB is nitrosylated primarily at Cys235 upon cell exposure to NO.

### Reduced AphB directly represses *hmpA* expression, and S-nitrosylation leads to inactive AphB

**Fig 2:**
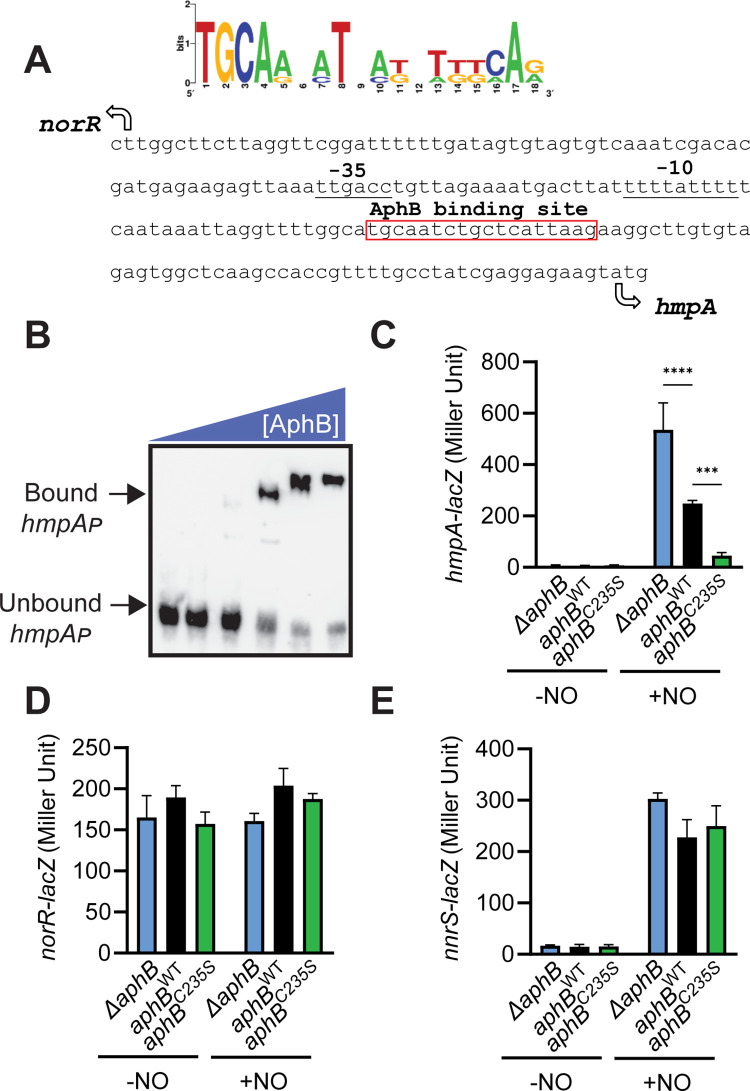
AphB S-nitrosylation and *hmpA* expression. **A.** AphB consensus binding site (curated in the CollecTF database: http://collectf.umbc.edu/browse/home/) analysis (generated by WebLogo: https://weblogo.berkeley.edu/logo.cgi) analysis revealed a putative binding site in the *hmpA* promoter region. Arrows: translational start. **B.** Gel shift assays using purified AphB-His_6_ and biotinylated *hmpA* promoter DNA. AphB-His_6_ proteins were added (from left to right) at the concentrations of 0, 0.125, 0.25, 0.5, 1, and 2 μM, respectively. 20 nM 50mer dsDNA containing the *hmpA* promoter region were used in each lane. **C-E.** The effects of AphB S-nitrosylation on the expression of NO-activated genes. *ΔaphB* with vector pBAD24, P_*BAD*_-*aphB*^WT^, or P_*BAD*_-*aphB*^C235S^ harboring *hmpA-lacZ* (**C**), *norR-lacZ* (**D**), or *nnrS-lacZ* (**E**) reporters were grown in the minimal medium containing 0.1% arabinose for 4 hrs microaerobically. When indicated, 100 μM DEA NONOate were then added, and all the cultures were incubated for an additional 2 hrs. β-galactosidase activity was then measured. Data are the means ± SD from three independent experiments. ***: P<0.0005; ****: P<0.0001 (two-way ANOVA).

AphB is a LysR-family transcription regulator that activates *V*. *cholerae* virulence gene expression. To study how S-nitrosylation of AphB may facilitate *V*. *cholerae* fitness under nitrosative stress, we first scanned for the consensus sequence motif of AphB binding (Fig 2A) across the *V*. *cholerae* genome. We identified an AphB box in the promoter region of *hmpA* (VCA0183), which encodes a key NO detoxifying enzyme [11]. To determine if AphB directly regulates the *hmpA* promoter, interaction was assessed by electrophoretic mobility shift assays (EMSA) using purified AphB and biotinylated *hmpA* promoter DNA fragments which contains the putative AphB binding site (Fig 2A). We found that the reduced form of AphB (in the presence of DTT) could bind the *hmpA* promoter (Fig 2B), and this binding was specific as inclusion of excess amount of unlabeled *hmpA* promoter DNA abolished the binding (S1A Fig). The AphB binding affinity to the *hmpA* promoter was ~50% lower than that of the *tcpP* promoter, a known target of AphB (S1B Fig). These data suggest that AphB may regulate *hmpA* expression directly.

Next, we examined how AphB regulates *hmpA*. We measured *hmpA-lacZ* expression in *ΔaphB* mutants containing an empty vector control, P_*BAD*_-*aphB*^WT^, and P_*BAD*_-*aphB*^C235S^. In the absence of NO, *hmpA* was not expressed, consistent with the previous report [11]. In the presence of NO, *hmpA* expression was highly induced in *ΔaphB* and *aphB*^WT^ strains but was low in the non-modifiable *aphB*^C235S^ mutant (Fig 2C). These data suggested reduced AphB represses *hmpA* expression. As *norR*, which encodes a transcriptional activator that turns on *hmpA* in the presence of NO, is divergently transcribed from *hmpA* (Fig 2A) [11], we examined whether AphB also regulates *norR* expression. By measuring the *norR-lacZ* activity in different *aphB* strain backgrounds, we found that *norR* expression was not controlled by either NO or AphB (Fig 2D). Moreover, since NorR-NO activates *nnrS* expression [11,12], we also examined whether AphB affects *nnrS* expression. Fig 2E shows that *nnrS* was induced by NO independent of AphB. Taken together, these data suggest that AphB represses *hmpA* expression directly and S-nitrosylation negatively affects AphB activity, as non-modifiable AphB^C235S^ mutants retained AphB repression of *hmpA* even in the presence of NO.

**Fig 3:**
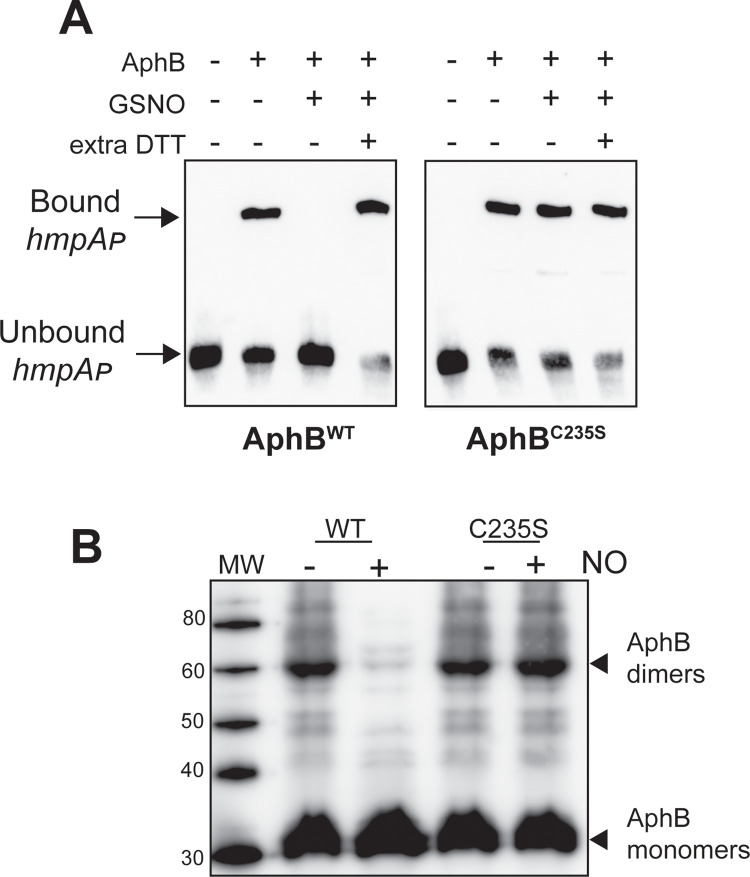
The effects of S-nitrosylation on AphB activity. **A.** S-nitrosylation *in vitro*. 1 μM of purified AphB^WT^ and AphB^C235S^ were incubated with or without 1 mM S-Nitroso-L-glutathione (GSNO) for 15 mins, and gel shift assays were performed. When indicated, extra 100 mM DTT was included in the binding reactions. **B.** The effect of NO on AphB dimerization. *ΔaphB* containing P_*BAD*_-*aphB*^WT^ or P_*BAD*_-*aphB*^C235S^ were grown in AKI medium for 4 hrs. 0.1% arabinose was then added. When indicated, 50 μM DEA NONOate was then added, and all the cultures were incubated for an additional 2 hrs. Cell pellets were then incubated with the crosslinking reagent DSP. The samples were then subjected to SDS-PAGE and western blots using anti-AphB antibody.

To further examine the effects of S-nitrosylation on AphB activity, we nitrosylated purified AphB^WT^ and AphB^C235S^ *in vitro* and tested their ability to bind to the *hmpA* promoter sequence. AphB^C235S^ proteins had similar binding affinity to the *hmpA* promoter as that of AphB^WT^ (Compare S2A and S2B Fig). When AphB^WT^ was treated with S-Nitroso-L-glutathione (GSNO), the AphB binding of *hmpA* was abolished (Fig 3A). Addition of DTT in the reaction restored AphB binding capacity. On the other hand, GSNO did not affect AphB^C235S^ binding of *hmpA* (Fig 3A, right panel). It has been shown that aerobic growth and oxidative stress lead to reduction of AphB dimerization, thus diminishing activity [5, 18]. To test whether nitrosative stress affects AphB dimerization as well, we conducted *in vivo* crosslink assays. Microaerobically cultured *V*. *cholerae* expressing AphB^WT^ and AphB^C235S^ with or without NO exposure was treated with the crosslinker DSP (dithiobis(succinimidyl propionate)) and western blot analyses were then performed. In the presence of NO, wildtype AphB dimer formation was significantly reduced, whereas similar numbers of dimers of AphB^C235S^ mutant proteins were formed without or with NO (Fig 3B). These data indicate that S-nitrosylation through the Cys235 residue reduces AphB multimerization.

### AphB S-nitrosylation is important for *V*. *cholerae* RNS resistance *in vitro* and *in vivo*

**Fig 4:**
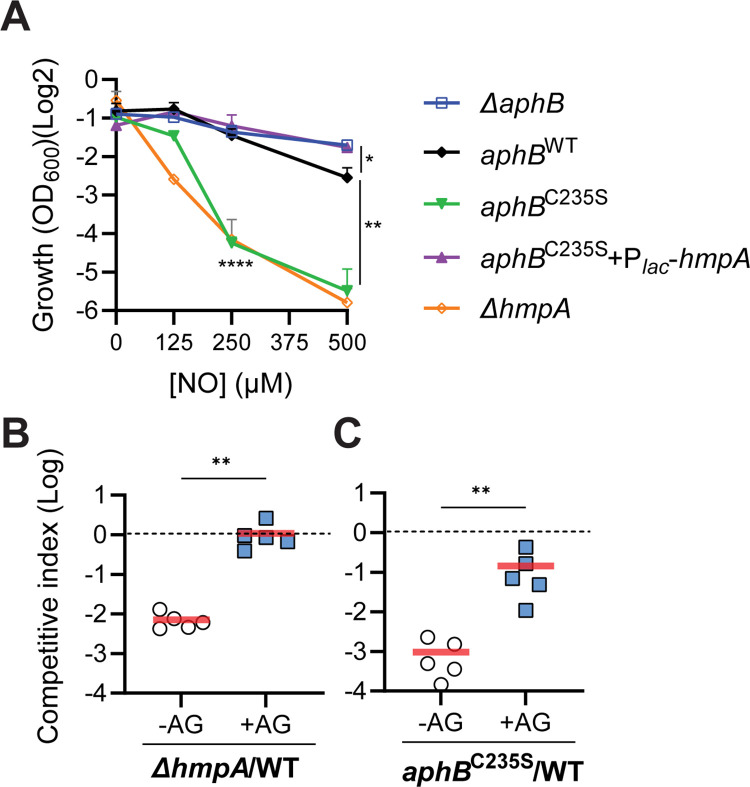
The effects of AphB S-nitrosylation on *V*. *cholerae* growth and colonization. **A.** NO resistance. Wildtype and *ΔhmpA* containing pBAD24 or *ΔaphB* with vector pBAD24, P_*BAD*_-*aphB*^WT^, or P_*BAD*_-*aphB*^C235S^ were grown in the M9 minimal medium containing 0.1% casein amino acids, 0.1% arabinose, 0.1 mM IPTG, and the different concentrations of DETA-NONOate indicated. When indicated, the strains harbored either pSRK-Gm or P_*tac*_-*hmpA* plasmids. The cultures were incubated at 37°C without shaking, and the OD_600_ was measured after 16 hrs. Data are the means ± SD from three independent experiments. *: P<0.05, **: P<0.01; ****: P<0.0001 (two-way ANOVA). **B & C.** Colonization. 10^8^ cells of wildtype and *ΔhmpA* mutants (**B**) or wildtype and *aphB*^C235S^ mutants (**C**) were mixed 1:1 and intragastrically administered to mice without aminoguanidine (AG) treatment (-AG) and mice with AG (+AG). At 7-day post-inoculation, *V*. *cholerae* colonization in the small intestine was determined. The competitive index (CI) was calculated as the ratio of mutant to wildtype colonies normalized to the input ratio. Horizontal line: mean CI of 5 mice. **: p <0.005 (Mann-Whitney U test).

Since AphB regulates the expression of *hmpA*, which encodes a key RNS resistance enzyme, next we investigated the relationship between AphB S-nitrosylation and RNS resistance. *V*. *cholerae* strains were grown in minimal media containing different concentrations of NO donors, and OD_600_ was then monitored. We found that compared to the wildtype, *aphB*^C235S^ mutants were more sensitive to NO, similar to that of *hmpA* deletion mutants, whereas *ΔaphB* mutants was slightly more resistant to NO (Fig 4A). Inclusion of a constitutively expressed *hmpA* in *aphB*^C235S^ mutants could relieve its sensitivity to NO (Fig 4A purple triangles), suggesting that AphB^C235S^ effects act through regulation of *hmpA*.

To examine the effect of S-nitrosylation on *V*. *cholerae* colonization, we used a streptomycin-treated adult mouse model, in which bacteria experience host-generated oxidative and nitrosative stress [11,19,20]. To mitigate host-derived RNS, we orally administrated aminoguanidine (AG), an iNOS inhibitor that is routinely used to reduce NO production in mice [21,22]. When the *ΔhmpA* mutant was co-inoculated with wildtype, *ΔhmpA* mutants displayed a significant colonization defect in both the small intestine (Fig 4B) and in feces (S3A Fig), consistent with the previous report [11]. When mice were treated with AG, however, *hmpA* mutant colonization was restored (Figs 4B and S3A) to a level similar to that of iNOS^-/-^ mice [11], confirming that *V*. *cholerae* uses HmpA to mitigate RNS and that AG treatment is sufficient to remove RNS stress *in vivo*. When the non-modifiable *aphB*^C235S^ mutants were tested in this mouse model, we found that in the -AG group, *aphB*^C235S^ mutants had an approximately 1000-fold reduction in colonization compared to the wildtype. Whereas in +AG group, *aphB*^C235S^ mutant colonization was only reduced by about 10-fold (Figs 4C and S3B). These data suggest that S-nitrosylation of AphB is important for *V*. *cholerae* to resist RNS during colonization.

### AphB repression of *hmpA* prevents intracellular ROS accumulation

**Fig 5:**
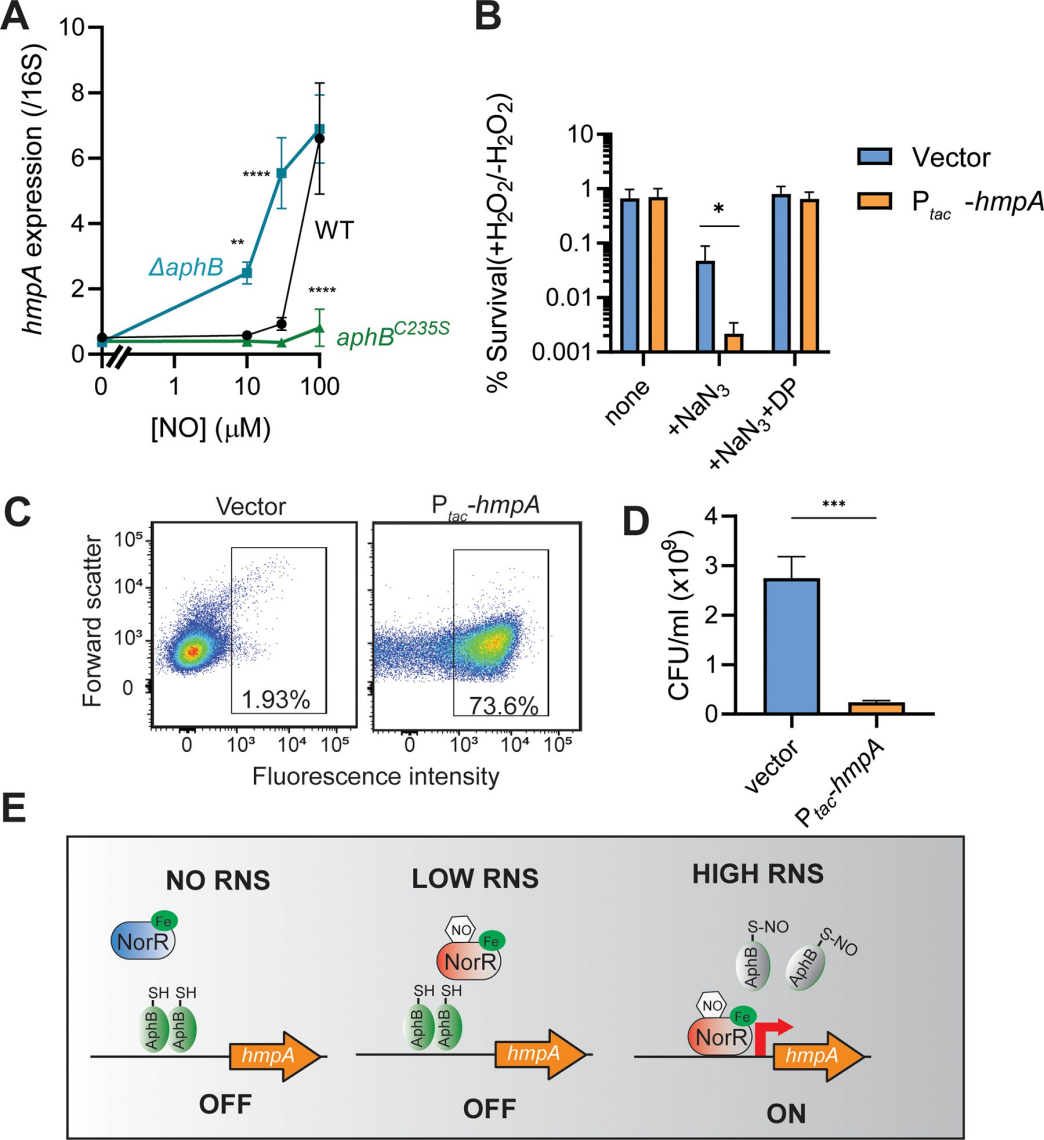
The physiological roles of AphB regulation of *hmpA*. **A.** AphB repression of *hmpA* at low NO concentrations. *ΔaphB* with vector pBAD24, P_*BAD*_-*aphB*^WT^, or P_*BAD*_-*aphB*^C235S^ were grown in the minimal medium containing 0.1% arabinose at 37°C microaerobically for 4 hrs. Various concentrations of DEA NONOate were added and incubated for an additional 1 hr. RNA was harvested and *hmpA* transcripts were quantified by qPCR and normalized against 16S rRNA. Data are the means ± SD from three independent experiments. **: p <0.001; ****: p <0.0001 (two-way ANOVA). **B.** Hypersusceptibility of HmpA-overexpressing cells to oxidative stress. 0.1% NaN_3_ and when indicated, 0.2 mM 2,2 -dipyridyl (DP) were added to the log-phase LB cultures for 30 mins. Cultures were then challenged with 2 mM H_2_O_2_ at 37°C for 30’. Viable counts were measured by serial dilution and plating onto LB agar. Data are the means ± SD from three independent experiments. *: p < 0.05 (Ordinary one-way ANOVA). **C&D.** HmpA overproduction on ROS accumulation (C) and growth (D). *V*. *cholerae* harboring an empty vector control or P_*tac*_*-hmpA* plasmid was grown in LB to stationary phase, and DCFDA staining was performed. Representative histograms of DCFDA-stained cells analyzed by using a CytoFLEX flow cytometer were shown in (C) and CFU counts were shown in (D). ***: p <0.0005 (Student t-test). **E.** Working model. Reduced AphB represses *hmpA* at low NO concentrations that may be enough to activate NorR. S-nitrosylation of AphB at high NO concentration leads to full induction of *hmpA*.

We next examined why AphB repression of *hmpA* is necessary, given that *hmpA* expression in the presence of NO is regulated by the transcriptional activator NorR (Fig 2C and [11]). We wondered if AphB repression of *hmpA* is important for *V*. *cholerae* fitness. To answer this question, we first compared *hmpA* transcription in wildtype and *ΔaphB* under different NO concentrations. In the wildtype, *hmpA* was induced by 100 μM NO, whereas in *ΔaphB* mutants, 10 μM NO could induce *hmpA* (Fig 5A). As a negative control, *hmpA* was not induced by NO in the *aphB*^C235S^ mutant (Fig 5A). These data suggest that AphB S-nitrosylation may be less sensitive to NO than that of NorR activation; therefore, AphB can still repress *hmpA* expression at low concentrations of NO.

Previous studies show that although HmpA flavohemoglobin plays an essential role in nitrosative stress responses in *E*. *coli* and *Salmonella*, dysregulation of *hmpA* may lead to the exacerbation of oxidative stress through Fenton reactions [23, 24]. Thus, we tested whether overexpression of HmpA affects *V*. *cholerae* susceptibility to hydrogen peroxide. Log-phase cultures were treated with sodium azide to inhibit respiration and subsequently challenged with H_2_O_2_. We found that the survival of HmpA-overexpressing cells was reduced more than 20-fold compared with cells harboring a vector (Fig 5B). Treatment with the iron chelators 2,2 -dipyridyl (DP) rescued the cells (Fig 5B), indicating that the HmpA-induced potentiation of oxidative stress susceptibility is iron-dependent. Furthermore, we measured intracellular reactive oxygen species (ROS) accumulation in stationary-phase cultures using the redox-sensitive, cell-permeable dye 2’,7’-dichlorodihydrofluorescein diacetate (DCFDA). We found that significant more ROS accumulated in HmpA-overexpressed cells (Fig 5C). The viability of HmpA-overexpressed cells was also reduced (Fig 5D). Taken together, these results suggest that AphB-mediated *hmpA* repression is important for *V*. *cholerae* fitness. We propose that reduced AphB plays an important role in the control of *hmpA* expression when NorR is already activated by NO at low concentrations. At high NO concentrations AphB is nitrosylated and therefore inactivated, which leads to NorR-activated *hmpA* expression to detoxify RNS (Fig 5E). In addition, when NO is removed, AphB may also play a role to prevent HmpA-mediated oxidative stress by faster restoration of repression of *hmpA* that is no longer needed. This hypothesis will be tested in a future study.

### AphB S-nitrosylation reduces virulence gene expression

**Fig 6:**
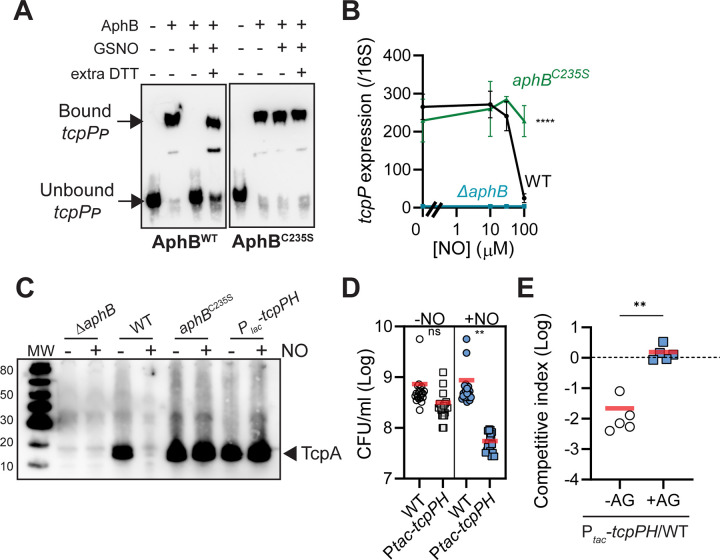
The impact of AphB S-nitrosylation on virulence. **A.** Interaction with the *tcpP* promoter. 1 μM of purified AphB^WT^ or AphB^C235S^ were incubated with or without 1 mM GSNO for 15 mins, and gel shift assays using a 50mer biotin-labeled dsDNA of *tcpP* were performed. When indicated, extra 100 mM DTT was included in the binding reactions. **B.** *tcpP* expression. *ΔaphB* with vector pBAD24, P_*BAD*_-*aphB*^WT^, or P_*BAD*_-*aphB*^C235S^ were grown in the minimal medium microaerobically for 3 hrs. Various concentrations of DEA NONOate were added and incubated for an additional 1 hr. RNA was harvested, and *tcpP* transcripts were quantified by qPCR and normalized against 16S rRNA. Data are the means ± SD from three independent experiments. ****: p <0.0001 (two-way ANOVA). **C.** TcpA production. *V*. *cholerae* strains were grown in the minimal medium microaerobically until mid-log phase. 0.1% arabinose (to induce *aphB*), 0.1 mM taurocholate (to induce virulence genes), and when indicated, 100 μM DEA NONOate were added and incubated for an additional 3 hrs. Cell lysates were then subjected to SDS-PAGE and western blot analysis using an anti-TcpA antibody. **D.** *In vitro* competition of a *tcpP* constitutive mutant. Wildtype and chromosomal *lacZ*::P_*tac*_*-tcpPH* mutants were cocultured in the AKI medium microaerobically for 8 hrs in the absence and in the presence of 100 μM DETA NONOate. CFU was then determined. Horizontal line: Mean of 20 repeats from three independent experiments. **: p <0.01 (Student’s t-test). **E.** *In vivo* competition. 10^8^ cells of wildtype and *lacZ*::P_*tac*_*-tcpPH* mutants were mixed 1:1 and intragastrically administered to mice without aminoguanidine (AG) treatment (-AG) and mice with AG (+AG). At 7-day post-inoculation, *V*. *cholerae* colonization in the small intestine was determined. The competitive index (CI) was calculated as the ratio of mutant to wildtype colonies normalized to the input ratio. Horizontal line: mean CI of 5 mice. **: p <0.005 (Mann-Whitney U test).

Since AphB is the key virulence activator that is required for *tcpP* expression, we next tested how AphB S-nitrosylation affects virulence gene expression. We first assessed the ability of AphB to bind to the *tcpP* promoter DNA by EMSA. We found that like the *hmpA* promoter (Fig 3A), S-nitrosylation abolished AphB^WT^, but not AphB^C235S^, binding of *tcpP* promoter (Fig 6A). Of note, AphB^C235S^ proteins had similar binding affinity to the *tcpP* promoter as that of AphB^WT^ (S2B Fig).

We then measured S-nitrosylation of AphB on *tcpP* transcription. We found that high concentrations of NO (100 μM) inhibited *tcpP* expression when wildtype *aphB* was present (Fig 6B, circles). In *aphB*^C235S^ mutants, however, *tcpP* was still expressed in the presence of NO (Fig 6B, triangles). As controls, *tcpP* was not induced in *ΔaphB* mutants (Fig 6B, squares). We then tested NO effects on downstream major virulence factor TcpA production by western blots. Fig 6C shows that little, if any, TcpA was produced in *ΔaphB* mutants. Addition of NO to wildtype significantly reduced TcpA production. However, NO did not affect TcpA when *aphB*^C235S^ was expressed or when a constitutively expressed *tcpP* was inserted on the chromosome (*lacZ*::P_*tac*_*-tcpPH*) to bypass the requirement of AphB activation. These data suggest that inactivation of AphB by S-nitrosylation leads to a reduction of virulence gene expression.

We then investigated whether the reduction of virulence via AphB S-nitrosylation could have a physiological role. We first performed growth competition assays in AKI medium with or without NO stresses using wildtype and the P_*tac*_-*tcpPH* strains. We found that the mutants displayed a slight growth defect in the absence of NO, but the viability of P_*tac*_-*tcpPH* strains was significantly reduced (Figs 6D and S3C). We then tested the P_*tac*_-*tcpPH* strain competitiveness *in vivo*. The mutant was defective in colonization of the gut of RNS-rich mice (-AG), but not in that of RNS-mitigated mice (+AG) (Figs 6E and S3C). These data suggest that it may be important for *V*. *cholerae* to reduce virulence gene expression when high RNS is encountered.

## Discussion

In this study, we found that when *V*. *cholerae* cells were exposed to nitric oxide, the key virulence regulator AphB was nitrosylated at Cys235, which inactivated AphB and allowed expression of the AphB-repressed *hmpA*. HmpA contains an iron-heme moiety that directly catalyzes the NO-decomposition reaction as well as a flavoreductase domain that mediates the transfer of the electrons to and from NO [25]. HmpA plays a critical role in *V*. *cholerae* RNS resistance *in vitro* and *in vivo* (Fig 4B and [11]). HmpA homologs are important for detoxification of NO during infection with other pathogenic bacteria, such as *E*. *coli*, *Yersinia pestis*, *Staphylococcus aureus*, and *Salmonella enterica* [23,26–28]. HmpA is also needed for *V*. *fischeri* initiation of symbiosis with squid hosts [29]. Previous studies show that in other pathogenic bacteria, several virulence regulatory proteins are nitrosylated under RNS exposure. For example, S-nitrosylation of SsrB Cys203, a two-component response regulator in *Salmonella* that activates *Salmonella* pathogenicity island-2 (SPI-2) type III secretion system, inhibits the DNA-binding capacity of SsrB, and exhibits increased fitness when exposed to RNS *in vivo* [30]. In *S*. *aureus*, S-nitrosylation of the quorum sensing (QS) regulator AgrA leads to decreased transcription of the *agr* operon and quorum sensing-activated toxin genes. In addition, in *E*. *coli*, nitrosylated OxyR activates genes that encode enzymes controlling S-nitrosylation and protecting against nitrosative stress under anaerobic conditions [31]. Interestingly, similar to that of AphB, OxyR also uses the reactive thiols to sense oxygen as well as ROS [32,33].

It is intriguing that *V*. *cholerae* deploys AphB to repress *hmpA*, which is only activated by NorR in the presence of NO [11]. In other pathogenic bacteria, such as *Salmonella*, HmpA production needs to be tightly regulated [23,24,34] because under aerobic conditions without nitrosative stress, elevated expression of *hmpA* increases bacterial susceptibility to ROS. We showed here that constitutive expression of *hmpA* in *V*. *cholerae* was detrimental to bacterial survival against ROS attacks or when bacteria were grown to stationary phase (Fig 5), suggesting the importance of AphB repression of *hmpA*. Interestingly, in almost all bacteria that harbor a *hmpA* gene, a NO-responsive repressor NsrR is involved in (or predicted to be) regulating *hmpA* [35]. These include Gram-positive bacilli and actinobacteria; β-proteobacteria such as *Ralstonia*, *Burkholderia*, *Bordetella*; γ-proteobacteria such as all members in *Enterobacteria*, *Shewanella*, all other *Vibrionales* except *V*. *cholerae*. The only exception is that in *Pseudomonas*, *hmpA* is predicted to be activated by NorR, as in the case of *V*. *cholerae*. NsrR belongs to the Rrf2 family of transcriptional regulators. It acts as a strong repressor of *hmpA* transcription and uses the iron-sulfur cluster to sense NO and relieve the repression of *hmpA* [36]. In general, repressors can reduce the basal level expression of a promoter and activators elicit high degree of induction [37], leading us to speculate that *V*. *cholerae* recruits AphB to serve as a repressor to perform the function of NsrR. It would be interesting to test if a similar repressor is present in other bacteria lacking NsrR, such as *Pseudomonas*, as well.

*V*. *cholerae* resides in both aquatic environments and in human small intestines. AphB plays a pivotal role in the life cycle of *V*. *cholerae*. During the transition from oxygen-rich environments to the oxygen-poor intestine, AphB Cys235 is reduced and can activate downstream virulence genes. The rate of reduction of AphB is low and another redox-sensing regulator, OhrR, is needed to jump-start virulence gene expression [38]. During ROS insults, AphB Cys235 is oxidized, which leads to the derepession of *ohrA*, encoding an organic hydroperoxide resistant protein [18]. This pathway of regulation can explain our data that *aphB*^C235S^ mutant colonization was not fully restored in the +AG mice (Fig 4C), as *ohrA* expression was repressed in *aphB*^C235S^ mutants. Similar to that of AphB sensing nitrosative stress we showed in this study, AphB Cys235 is less sensitive to oxidation than that of OhrR oxidation, thus OhrA production is tightly regulated. Evidently, AphB Cys235 is less prone to redox potential changes, and this may be important for *V*. *cholerae* physiology.

AphB is a key virulence regulator. S-nitrosylation of AphB leads to the reduction of *tcpPH* expression and downstream TcpA production (Fig 6). An inability to turn off *tcpPH* expression in the presence of nitrosative stress resulted in growth disadvantages *in vitro* and *in vivo* (Fig 6). The mechanisms behind this are currently unknown. We tested *tcpA* deletion in P_*tac*_*-tcpPH* strain and found that it still displayed the growth defect with NO (S4 Fig), suggesting that dysregulated TcpA pilin production is not involved in the RNS resistance. Interestingly, *V*. *cholerae* employs QS systems to temporally control virulence during infection. QS represses virulence gene expression and biofilm formation while activating production of extracellular proteases, suggesting the importance of QS in entering and exiting the host [39–42]. Host-derived RNS increases as the infection progresses [8], which increases the rate of detachment from the epithelium and subsequently could permit individual cells to establish new infection foci in the intestine or to exit the host. AphB S-nitrosylation therefore provides another layer of regulation to repress virulence as needed.

## Methods and materials

### Ethics statement

All animal experiments were performed in strict accordance with the animal protocol (Protocol No. 805287) that was approved by the Institutional Animal Care and Use Committee (IACUC) of the University of Pennsylvania.

### Strains, plasmids, and culture conditions

*V*. *cholerae* El Tor C6706 [43] was used as the primary wildtype strain in this study. Both *V*. *cholerae* and *E*. *coli* were propagated in LB (Luria-Bertani) Miller medium with appropriate antibiotics at 37°C, unless otherwise noted. AKI medium was used to induce virulence gene expression [44]. Cultures were grown aerobically (shaking at 250 rpm) or microaerobically (stationary growth). For supplying NO donors in cultures, either diethylammonium (Z)-1-(N,N-diethylamino)diazen-1-ium-1,2-diolate (DEA NONOate)(half-life of 2 minutes at 37°C) or (Z)-1-[N-(2-aminoethyl)-N-(2-ammonioethyl)amino]diazen-1-ium-1,2-diolate (DETA NONOate)(half-life of 20 hours at 37°C) (Cayman Chemical) was used.

In-frame deletion of *aphB* (VC1049), P_*BAD*_-*aphB* wildtype and cysteine to serine mutant derivatives, and plasmids to make recombinant AphB proteins (AphB-His_6_) were constructed as described previously [5]. Transcriptional *hmpA*- (VCA0183), *nnrS*- (VC2330), and *norR*- (VCA0182) LacZ reporters, and *hmpA* in-frame deletion were constructed as described in [11]. P_*tac*_*-hmpA* plasmid was constructed by cloning *hmpA* coding sequences into pMal-c2x (New England Biolab) and then subcloned to pSRK-Gm [45]. Chromosomal *aphB* variants used for mouse experiments were constructed by the Tn7-based genomic integration system [46]. Briefly, DNA sequences of *aphB* wildtype or C235S mutant with the native promoter (341 bp upstream of ATG) were PCR amplified and cloned into the pGP704-mTn7 vector via NotI restriction cloning. The insertion of the *aphB* wildtype or C235S mutant into the *V*. *cholerae* genome was done by triparental mating among *V*. *cholerae* Δ*aphB*, *E*. *coli* S17-1 λpir carrying the helper plasmid pUX-BF13, and *E*. *coli* SM10 λpir carrying *aphB* variants on pGP704-mTn7. Exconjugants were selected on thiosulfate-citrate-bile salts-sucrose (TCBS) agar plates containing gentamicin (20 μg/ml). The insertion of mini Tn7 cassette at the intergenic region of VC0487 and VC0488 was confirmed by PCR and sequencing using flanking primers of insertion site. The chromosomal *lacZ*::P*tac-tcpPH* strain was constructed by the multiplex genome editing by natural transformation (MuGENT) method [47]. Briefly, P*tac* promoter and *tcpPH* sequence were PCR amplified from pMal-c2x (New England Biolab) and *V*. *cholerae* C6706 genome respectively, stitched with the *lacZ* UP and Down 3kb arms, cotransformed into *V*. *cholerae* C6706 with VC1807::spec DNA, and selected on LB agar plates containing spectinomycin (100 μg/ml). The insertion of P*tac-tcpPH* was confirmed by PCR and sequencing. The primer sequences used for the study and further details about the constructions are available upon request.

### Proteomic profiling of thiol S-nitrosylation in *V*. *cholerae*

Iodoacetyl isobaric tandem mass tags (iodoTMT) labeling of S-nitrosylated thiols was performed by modifying the method described previously [14,48]. Briefly, *V*. *cholerae* were grown in AKI medium without shaking for 4 hrs and then challenged with 100 μM DEA NONOate for 1 hr. The cell lysates were first incubated with 20 mM methyl methanethiosulfonate (MMTS) (Pierce) and then reduced with 20 mM sodium ascorbate. Proteins containing reduced thiols were then labeled with iodoTMTzero and digested with trypsin (8 μg trypsin/100 μg proteins). IodoTMTzero-labeled peptides were enriched by using the Anti-TMT Resin and eluted by TMT elution buffer (Thermo Scientific). The eluted peptides were submitted to Poochon Scientific (Frederick, MD). Liquid chromatography tandem mass spectrometry (LC-MS/MS) analysis was performed using a Q Exactive Orbitrap mass spectrometer (Thermo Scientific) coupled with an UltiMate 3000 nano UPLC system (Thermo Scientific). Peptide sequences were identified using the Proteome Discoverer 2.4 software (Thermo, San Jose, CA) based on the SEQUEST and percolator algorithms. MS/MS spectra were searched against a UniProt *V*. *cholerae* protein database and a common contaminants database using full tryptic specificity with up to two missed cleavages. Variable modifications included in the search were an addition mass of TMTzero (324.2 Da) on cysteine. Consensus identification lists were generated with false discovery rates set at 1% for protein, peptide, and site identifications.

### Detection of S-nitrosylated AphB using biotin switch assays

Overnight cultures of *ΔaphB* with P_*BAD*_-*aphB*^WT^, P_*BAD*_-*aphB*^C76S/C94S^, and P_*BAD*_-*aphB*^C235S^ were inoculated into the AKI medium and incubated microaerobically at 37°C for 4 hrs. 0.1% arabinose and, when indicated, 100 μM DEA-NONOate were added and further incubated for 1 hr. Bacterial cells were collected and subjected to Cayman Chemical’s S-nitrosylated protein detection assay kit (biotin switch) [17] with modifications. Briefly, total proteins were treated with MMTS to block free thiols, reduced by sodium ascorbate, and labeled with biotin. Biotinylated proteins were then enriched by using avidin agarose (Pierce). Proteins from both flowthrough and eluted samples were separated by sodium dodecyl sulfate-polyacrylamide gel electrophoresis (SDS-PAGE) and AphB was detected by western blot using an anti-AphB antibody [5].

### Gel retardation assays

AphB^WT^-His_6_ and AphB^C235S^-His_6_ proteins were expressed and purified on nickel columns according to the manufacturer’s instructions (Qiagen). Double-stranded biotin-labeled *hmpA* (CAATAAATTAGGTTTTGGCATGCAATCTGCTCATTAAGAAGGCTTGTGTAGAGTGGCTCA) and *tcpP* (GCAATTAAGTTCTCATTATCAACTGCAGAATTAGATTGCAAATAATTATATTAAAAAAAA) probes were prepared by annealing the single-stranded 5’biotin-labeled sense and antisense 50mer oligos after denaturation. Binding reactions contained various amount of AphB proteins and 20 nM of DNA in a buffer consisting of 10 mM Tris-HCl (pH 7.5), 1 mM EDTA, 50 mM KCl, 100 μg/ml BSA, 10 μg of poly dI-dC/ml, and when indicated, 100 mM dithiothreitol (DTT). After 20 minutes of incubation at 25°C, samples were size-fractionated using 5% polyacrylamide gels in 0.5X Tris-acetate-EDTA (TAE) buffer. The biotin-labeled free DNA and protein-DNA complexes were electrophoretic transferred via binding reactions to nylon membrane and visualized using horseradish peroxidase (HRP) conjugated streptavidin (Pierce). To S-nitrosylate purified AphB proteins, DTT was first removed from AphB^WT^ and AphB^C235S^ by dialysis. 1 μM proteins were then incubated with 1 mM S-Nitroso-L-glutathione (GSNO) (Cayman Chemical) for 15 mins at 25°C.

### Gene expression studies

Overnight cultures of *ΔaphB* with vector pBAD24, P_*BAD*_-*aphB*^WT^, or P_*BAD*_-*aphB*^C235S^ harboring different *lacZ* reporters were inoculated 1:100 into the minimal medium described in [11] containing 0.1% arabinose. After 4 hrs of growth, various amounts of DEA-NONOate indicated were added to the cultures. Two hours later, β-galactosidase activity was measured. For qPCR quantification of *hmpA* and *tcpP* transcripts, the above cultures were treated with DEA-NONOate for one hour before bacterial cells were harvested and total RNA was purified by using RNeasy Kit (Qiagen). RNA reverse transcription was performed by using the SuperScript II Kit (Invitrogen). Quantitative realtime qPCR was performed on a CFX96 Real-Time system (BioRad) using primers specific for *hmpA* and *tcpP*. The 16S rRNAs were used as internal controls in all reactions.

### Detection of TcpA and AphB

Whole-cell lysates were prepared from bacterial cultures and samples were normalized to the amount of total protein as assayed by the Biorad protein assay (Biorad). The samples were separated by sodium dodecyl sulfate-polyacrylamide gel electrophoresis (SDS-PAGE) on a 12% polyacrylamide gel and transferred to nitrocellulose membrane for western blot analysis using polyclonal rabbit anti-TcpA [40] or AphB antibody[5].

### *In vivo* crosslinking of AphB

*ΔaphB* containing P_*BAD*_-*aphB*^WT^ or P_*BAD*_-*aphB*^C235S^ plasmids were grown in the AKI medium for 4 hrs. 0.1% arabinose and when indicated, 50 μM DEA NONOate were then added and all the cultures were incubated for an additional 2 hrs. Cells were resuspended in PBS and treated with 0.8 mM crosslinking reagent DSP (dithiobis(succinimidyl propionate))(Thermo Fisher) on ice for 2 hrs. The cross-linking reactions were terminated by adding 50 mM Tris-HCl (pH 7.5). Cell pellets were resuspended in SDS loading buffer without reducing agents and AphB multimers were detected by western blots using AphB antibody.

### Intracellular ROS accumulation

*V*. *cholerae* containing a vector control or P_*tac*_-*hmpA* plasmid were grown in LB with 0.5 mM IPTG at 37°C for 8 hrs with aeriation. Cell pellets were washed with PBS and resuspended in PBS with 20 μM of the redox-sensitive, cell-permeable dye 2’,7’-dichlorodihydrofluorescein diacetate (DCFDA) (Abcam). Cells were then stained for 1 hr at 37°C, and DCFDA-stained cells were subjected to flow cytometry (CytoFLEX, BD) for ROS detection using the 488 nm laser for excitation and detected at 535 nm.

### *V*. *cholerae* mouse colonization

The streptomycin-treated adult mouse model was used to examine *V*. *cholerae* RNS resistance *in vivo*. Briefly, six-week-old CD-1 mice were used. 0.5% (wt/vol) streptomycin and 0.5% aspartame were added to the drinking water throughout the experiment. After 3 days of streptomycin treatment, 1 mg/ml aminoguanidine (AG, Acros Organics) was added to the drinking water of +AG group of mice and throughout remainder of the experiment. Additionally, the equivalent of 1 mg of AG was orally gavaged into each mouse in the +AG group of mice daily throughout the remainder of the experiment. One day after starting the AG treatment, approximately 10^8^ CFU of each of the two differentially labeled strains (wildtype *lacZ*^+^ and mutant l*acZ*^-^) were mixed at a 1:1 ratio and intragastrically administered to each mouse. Fecal pellets were collected from each mouse at the indicated time points, resuspended in LB, serially diluted, and then plated on plates containing 5-bromo-4-chloro-3-indolyl-β-D-galactopyranoside (X-gal) and appropriate antibiotics. At day 7, small intestine tissues were harvested and CFU of small intestine-colonized *V*. *cholerae* were enumerated. The competitive index was calculated as the ratio of mutant to wildtype colonies normalized to the input ratio.

## Supporting information

S1 Fig EMSA controls. **A.** Specificity of AphB binding of *hmpA*. AphB-His6 proteins were mixed with 20 nM 50mer biotin-labeled double-stranded *hmpAp* DNA in the absence or in the presence of 1 μg unlabeled *hmpA* promoter DNA. **B.** AphB on *tcpP*. AphB-His_6_ proteins added (from left to right) at concentrations of 0, 0.125, 0.25, 0.5, 1, and 2 μM, respectively. 20 nM 50mer dsDNA containing the *tcpP* promoter region were used in each lane.
(PDF)

S2 Fig EMSA using purified AphB^C235S^-His_6_. AphB-His_6_ proteins added (from left to right) at concentrations of 0, 0.125, 0.25, 0.5, 1, and 2 μM, respectively. 20 nM 50mer dsDNA containing *hmpA* (**A**) or *tcpP* (**B**) promoter region were used in each lane.
(PDF)

S3 Fig Streptomycin-treated adult mouse colonization. 10^8^ cells of wildtype and *ΔhmpA* mutants (**A**) or wildtype and *aphB*^C235S^ mutants (**B**) or wildtype and P_*tac*_*-tcpPH* mutants (**C**) were mixed 1:1 and intragastrically administered to mice without aminoguanidine (AG) treatment (-AG) and mice with AG (+AG). *V*. *cholerae* CFU from fecal samples was determined daily. The competitive index (CI) was calculated as the ratio of mutant to wildtype colonies normalized to the input ratio. Horizontal line: mean CI of 5 mice. *: p <0.05; **: p <0.005; ***: p <0.001 (Mann-Whitney U test).
(PDF)

S4 Fig TcpA effects in the *tcpP* constitutive mutant. Wildtype and *lacZ*::P_*tac*_*-tcpPH* mutants or wildtype and *lacZ*::P_*tac*_*-tcpPH/ΔtcpA* mutants were cocultured in the AKI medium microaerobically for 8 hrs in the absence and in the presence of 100 μM DETA NONOate. CFU was then determined. Data are the means ± SD from 6 independent experiments. ***: p <0.005 (Ordinary one-way ANOVA).
(PDF)

S1 Dataset *V*. *cholerae* S-nitrosylation proteome. (XLSX)
