# Supplementary material for: S-Nitrosylation of the virulence regulator AphB promotes Vibrio cholerae pathogenesis
Source: PLoS Pathog. 2022 Jun 17;18(6):e1010581. doi: 10.1371/journal.ppat.1010581 (PMC9246220; doi:10.1371/journal.ppat.1010581)
Supplement: S1 Fig — A. Specificity of AphB binding of hmpA. AphB-His6 proteins were mixed with 20 nM 50mer biotin-labeled double-stranded hmpAp DNA in the absence or in the presence of 1 μg unlabeled hmpA promoter DNA. B. AphB on tcpP. AphB-His6 proteins added (from left to right) at concentrations of 0, 0.125, 0.25, 0.5, 1, and 2 μM, respectively. 20 nM 50mer dsDNA containing the tcpP promoter region were used in each lane. (PDF) [file ppat.1010581.s001.pdf]

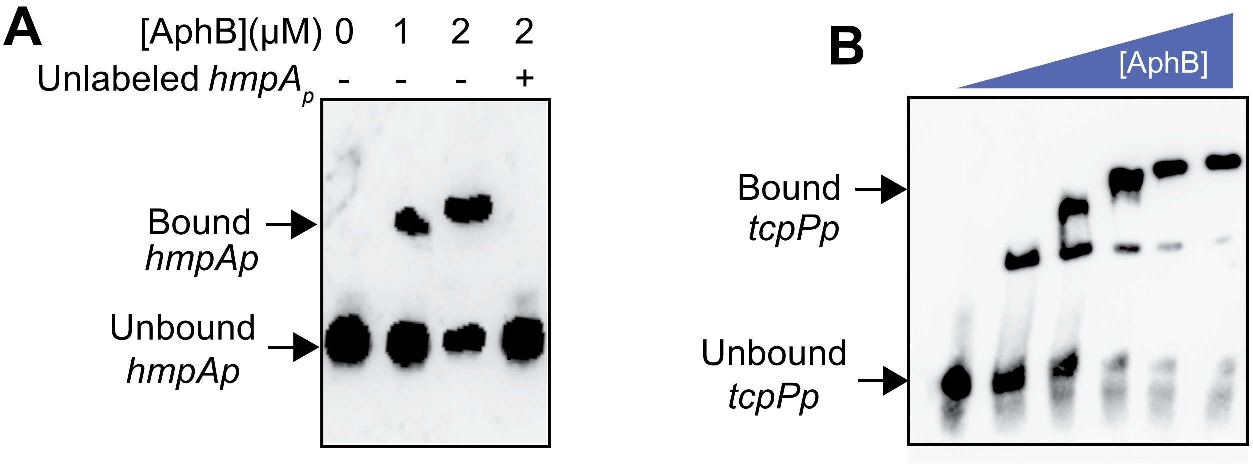

Fig. S1. EMSA controls. A. Specificity of AphB binding of *hmpA*. AphB-His6 proteins were mixed with 20 nM 50mer biotin-labeled double-stranded *hmpAp* DNA in the absence or in the presence of 1 μg unlabeled *hmpA* promoter DNA. **B.** AphB on *tcpP*. AphB-His_6 proteins added (from left to right) at concentrations of 0, 0.125, 0.25, 0.5, 1, and 2 μM, respectively. 20 nM 50mer dsDNA containing the *tcpP* promoter region were used in each lane.
