# Supplementary material for: S-Nitrosylation of the virulence regulator AphB promotes Vibrio cholerae pathogenesis
Source: PLoS Pathog. 2022 Jun 17;18(6):e1010581. doi: 10.1371/journal.ppat.1010581 (PMC9246220; doi:10.1371/journal.ppat.1010581)
Supplement: S2 Fig — AphB-His6 proteins added (from left to right) at concentrations of 0, 0.125, 0.25, 0.5, 1, and 2 μM, respectively. 20 nM 50mer dsDNA containing hmpA (A) or tcpP (B) promoter region were used in each lane. (PDF) [file ppat.1010581.s002.pdf]

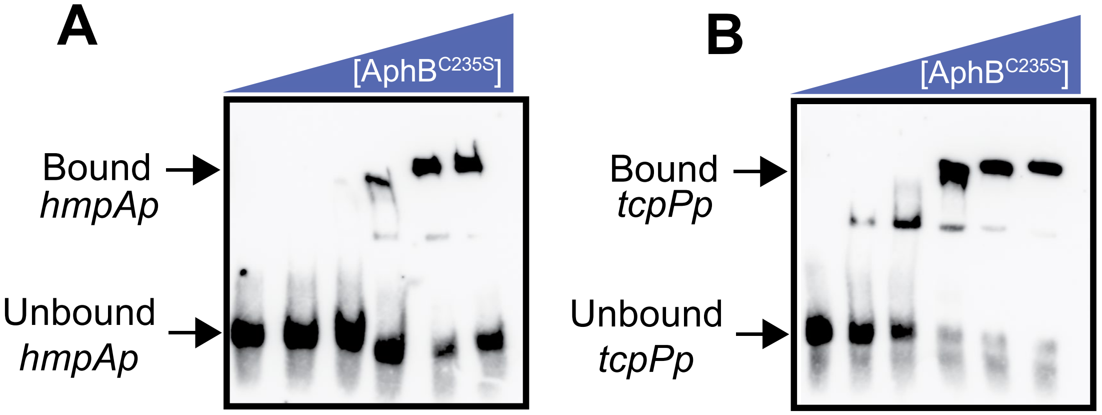

Fig. S2. EMSA using purified AphB^{C235S}-His$_6$. AphB-His$_6$ proteins added (from left to right) at concentrations of 0, 0.125, 0.25, 0.5, 1, and 2 µM, respectively. 20 nM 50mer dsDNA containing *hmpA* (**A**) or *tcpP* (**B**) promoter region were used in each lane.
