# Supplementary material for: S-Nitrosylation of the virulence regulator AphB promotes Vibrio cholerae pathogenesis
Source: PLoS Pathog. 2022 Jun 17;18(6):e1010581. doi: 10.1371/journal.ppat.1010581 (PMC9246220; doi:10.1371/journal.ppat.1010581)
Supplement: S3 Fig — 108 cells of wildtype and ΔhmpA mutants (A) or wildtype and aphBC235S mutants (B) or wildtype and Ptac-tcpPH mutants (C) were mixed 1:1 and intragastrically administered to mice without aminoguanidine (AG) treatment (-AG) and mice with AG (+AG). V. cholerae CFU from fecal samples was determined daily. The competitive index (CI) was calculated as the ratio of mutant to wildtype colonies normalized to the input ratio. Horizontal line: mean CI of 5 mice. *: p <0.05; **: p <0.005; ***: p <0.001 (Mann-Whitney U test). (PDF) [file ppat.1010581.s003.pdf]

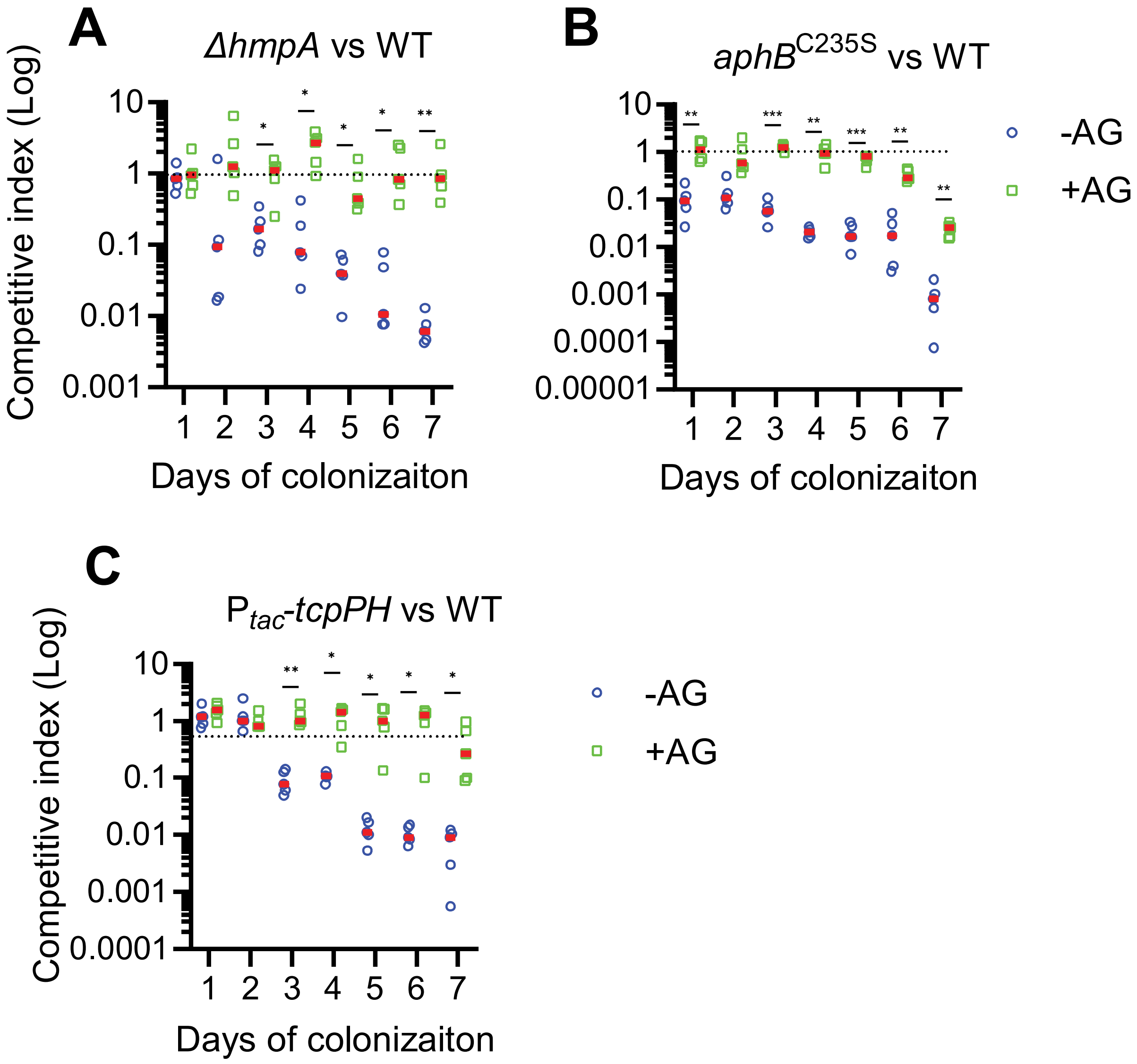

Fig. S3. Streptomycin-treated adult mouse colonization. 10^8 cells of wildtype and Δ*hmpA* mutants (**A**) or wildtype and *aphB*^{C235S} mutants (**B**) or wildtype and P$_{tac}$-*tcpPH* mutants (**C**) were mixed 1:1 and intragastrically administered to mice without aminoguanidine (AG) treatment (-AG) and mice with AG (+AG). *V. cholerae* CFU from fecal samples was determined daily. The competitive index (CI) was calculated as the ratio of mutant to wildtype colonies normalized to the input ratio. Horizontal line: mean CI of 5 mice. *: p <0.05; **: p <0.005; ***: p <0.001 (Mann-Whitney U test).
