# Supplementary material for: S-Nitrosylation of the virulence regulator AphB promotes Vibrio cholerae pathogenesis
Source: PLoS Pathog. 2022 Jun 17;18(6):e1010581. doi: 10.1371/journal.ppat.1010581 (PMC9246220; doi:10.1371/journal.ppat.1010581)
Supplement: S4 Fig — Wildtype and lacZ::Ptac-tcpPH mutants or wildtype and lacZ::Ptac-tcpPH/ΔtcpA mutants were cocultured in the AKI medium microaerobically for 8 hrs in the absence and in the presence of 100 μM DETA NONOate. CFU was then determined. Data are the means ± SD from 6 independent experiments. ***: p <0.005 (Ordinary one-way ANOVA). (PDF) [file ppat.1010581.s004.pdf]

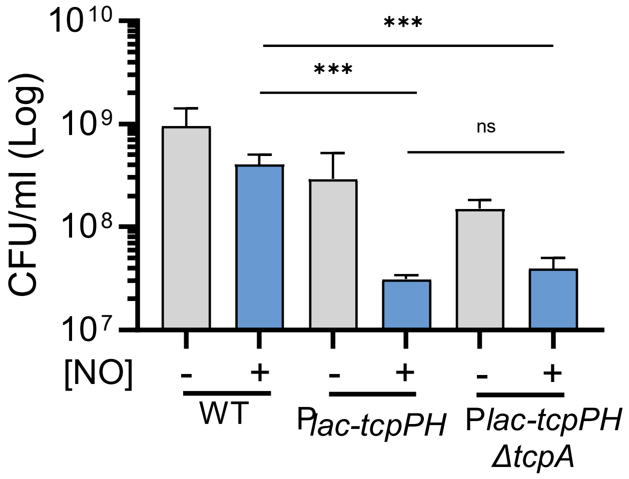

Fig. S4. TcpA effects in the *tcpP* constitutive mutant. Wildtype and *lacZ*::P$_{tac}$-*tcpPH* mutants or wildtype and *lacZ*::P$_{tac}$-*tcpPH*/Δ*tcpA* mutants were cocultured in the AKI medium microaerobically for 8 hrs in the absence and in the presence of 100 μM DETA NONOate. CFU was then determined. Data are the means ± SD from 6 independent experiments. ***: p <0.005 (Ordinary one-way ANOVA).
